# Effects of blood glucose and glycosylated hemoglobin levels on intravenous thrombolysis in patients with acute cerebral infarction and type 2 diabetes mellitus

**DOI:** 10.12669/pjms.35.3.8

**Published:** 2019

**Authors:** Zhaoting Zhang, Mingyue Qian, Zhonglin Ge, Ping Zhou, Jianhua Liu, Jiechun Chen

**Affiliations:** 1 *Zhaoting Zhang, The Second People’s Hospital of Lianyungang, Lianyungang, Jiangsu, PR China*; 2 *Mingyue Qian, The Second People’s Hospital of Lianyungang, Lianyungang, Jiangsu, PR China*; 3 *Zhonglin Ge, The Second People’s Hospital of Lianyungang, Lianyungang, Jiangsu, PR China*; 4 *Ping Zhou, The Second People’s Hospital of Lianyungang, Lianyungang, Jiangsu, PR China*; 5 *Jianhua Liu, The Second People’s Hospital of Lianyungang, Lianyungang, Jiangsu, PR China*; 6 *Jiechun Chen, The Second People’s Hospital of Lianyungang, Lianyungang, Jiangsu, PR China*

**Keywords:** Acute Cerebral Infarction, Glycosylated Hemoglobin, Intravenous Thrombolysis

## Abstract

**Objective:**

To investigate the effect of fasting plasma glucose (FPG) and glycosylated hemoglobin (HbAlc.) levels on thrombolytic therapy in patients with acute cerebral infarction and type 2 diabetes mellitus.

**Methods:**

A total of 135 patients with acute cerebral infarction were selected for this study. They were divided into study group (n=70, with acute cerebral infarction & type 2 diabetes mellitus) and control group (n=65, with acute cerebral infarction but no type 2 diabetes mellitus). All patients underwent thrombolysis treatment with Alteplase for injection. The patients were evaluated by the national institutes of health stroke scale (NIHSS) score, the modified Rankin scale (MRS) score and the Barthel index score, such indicators in patients as FPG, HbAlc, triglyceride (TG), low density lipoprotein cholesterol (LDL-C), total cholesterol (TC) and high density lipoprotein cholesterol (HDL-C) were determined, the fast blood sugar before thrombolysis and the treatment effect after 24h thrombolysis in the observation group were observed and meanwhile the mortality rate in patients after 5 months thrombolysis was analyzed.

**Results:**

Compared with before thrombolysis, the indexes of the two groups were significantly improved after thrombolysis, and the improvements of FPG, HbAlc, TG and LDL-C in the control group were better than those in the study group (P<0.05). There was no significant difference between the two groups in the levels of TC and HDL-C after thrombolysis (P>0.05). The 24h MBG, SDBG and MAGE in the study group were higher than those in the control group (P<0.05). In the study group, when the blood glucose was less than 6.0mmol/L before thrombolysis, the lowest effective rate after 24h thrombolysis was 33.3%, and when the blood glucose was ranging from 7.0 to 9.0mmol/L, the highest effective rate after 24h thrombolysis was 73.9%, and with the gradual increase of blood glucose, the effective rate after 24h thrombolysis decreased gradually. Also the effective rate after 24h thrombolysis also decreased gradually with the increase of HbAlc value, it reached the highest value of 64.4% at HbAlc <6.0mmol/Lad the lowest value of 25% at HbAlc >7.0mmol/L. Compared with the control group, the MHSS score and MRS score were higher and the Barthel index after thrombolysis was lower in the study group with the difference being statistically significant (P<0.05). The five months mortality rate after thrombolytic therapy was 12.9% (9/70) in the study group and 10.8% (7/65) in the control group, with no significant difference between the two groups (P=0.316). The incidence of intracranial hemorrhage after thrombolytic therapy was higher in the study group than in the control group, but the difference was not statistically significant (P>0.05), however there was significant difference between the two groups in revascularization and prognosis (P<0.05).

**Conclusion:**

The level of HbAlc affected the curative efficacy, the higher the level, the poorer the efficacy and to control the blood glucose within a certain range before thrombolysis was beneficial to enhance the effect of static thrombolysis.

## INTRODUCTION

Diabetes has got increasingly high incidence rate year by year and became a worldwide disease as well as a public health problem. Diabetes mellitus is an independent risk factor of first stroke and also an important factor affecting the prognosis of cerebral infarction.^1^ China is one of the countries with the highest incidence and mortality rate of ischemic stroke in the world. The disease of cerebral infarction has caused great losses and heavy burdens for the family, society and the country. Acute cerebral infarction is one of the most common and serious complications of diabetes mellitus.^2^ Its incidence in diabetic patients is 2.5-3.5 times higher than that in non-diabetic patients. According to statistics^3^, about 20% of patients with acute cerebral infarction have diabetes. It has worse prognosis in diabetic patients than in those without diabetes history. Intravenous thrombolysis is one of the methods confirmed to be effective in acute cerebral infarction. The current guidebook to ultra-early intravenous thrombolysis for cerebral infarction strict rules on the blood glucose of patients conforming to time windows because the over high blood sugar will increase the risk of intravenous thrombolysis and affect the prognosis.^4^,^5^ There are few studies about the effect of blood glucose fluctuation on thrombolytic therapy for cerebral infarction.

Our objective was to investigate the effect of fasting plasma glucose (FPG) and glycosylated hemoglobin (HbAlc.) levels on thrombolytic therapy in patients with acute cerebral infarction and type 2 diabetes mellitus.

## METHODS

We selected 135 patients with acute cerebral infarction admitted to our hospital from January 2016 to May 2017 for this study.

### Inclusion Criteria

1. Patients were aged 20-80
2. The signs of brain function impairment persisted more than 30 minutes and the National Institutes of Health Stroke Scale (NIHSS) score was 4-24.
3. The disease time was within 4.5h.
4. Patients were in line with WHO diagnostic criteria for diabetes, 1999.

### Exclusion Criteria

1. Patients with cerebral hemorrhage or bleeding tendency of other systems.
2. Patients with dysfunction of organs such as heart and liver.
3. Women during pregnancy and lactation.
4. Patients with allergic constitution.

The selected patients were divided into the group consisting of 70 patients with acute cerebral infarction and type 2 diabetes mellitus (study group) and the group consisting of 65 patients with acute cerebral infarction but no type 2 diabetes mellitus (control group). In the study group there were 40 male and 30 female aged 35-74 (64.2±5.5) years and with a history of diabetes for 2-16 years, including 42 cases with hypertension and 28 with hyperlipidemia. In the control group, there were 38 male and 27 female aged 34-75 (63.5±4.7) years, including 38 with hypertension and 27 with hyperlipidemia. There was no significant difference between the two groups in patients gender, age and cerebral infarction status (P>0.05), suggestive of comparability.

All patients were given thrombolysis therapy with Alteplase for Injection (rt-PA) freeze-dry powder (provided by Beijing Aide Pharmaceutical Co., Ltd.)., ten percent of which was diluted at the total dose of 0.9mg/kg with physiological saline (10ml) within three hours of symptoms emerging followed by peripheral intravenous injection, after 1min, the remaining 90% was diluted with physiological saline (250 ml) followed by continuous injection for 60min through infusion pump, the maximum treatment dose being 90mg. At 24h after thrombolytic therapy, patients were not given aspirin or clopidogrel and those with diabetes or stress-induced hyperglycemia after cerebral infarction underwent glucose-lowering treatment with insulin, which was forbidden in patients with severe stroke (by clinical diagnosis or imaging diagnosis) and with the basic value of blood glucose under 2.78mmol/L or over 22.22mmol/L.

### Observation Index

1. The patients were extracted 10ml venous blood at admission and after 24h thrombolysis, which was vetted by clinical laboratory for determinations of such indicators as plasma glucose (PG), glycosylated hemoglobin (HbAlc), triglycerides (TG), total cholesterol (TC), high density lipoprotein cholesterol (HDL-C) and low density lipoprotein the cholesterol (LDL-C).
2. The evaluation of indexes for dynamic blood glucose:
  (a)Mean blood glucose (MBG): the normal value was under 6.6mmol/L;
  (b)Standard deviation of blood glucose (SDBG): its assessment deviates from the value of average blood glucose on the whole, reflecting the discrete characteristics of glucose, but unable to distinguish between the main and minor fluctuations with its normal value under1.4mmol/L;
  (c)Mean amplitude of glycemic excursions(MAGE): the glucose fluctuation with the amplitude no more than 1 SDBG was set as effective fluctuation, according to direction of the first effective fluctuation, the average value was obtained as the “gold standard” for reflection of blood glucose fluctuation, the normal value being MAGE <3.9mmol/L.
3. The NIHSS score, MRS score and Barthel index score were assessed. The high NIHSS and MRS scores and low Barthel index score indicate poor recovery effect after thrombolytic therapy.
4. The five months mortality after thrombolytic therapy.

### Statistical Methods

SPSS 21 software was used for statistical analysis. Enumeration data were represented by rate (%) and assessed by *x*^2^ test, and measurement data were expressed by mean ±standard deviation, and assessed by t test. P<0.05 suggested there was statistically significant difference.

### Ethical Consideration

Tis study was approved from the institutional ethical review board of The Second People`s Hospital of Lianyungang, Lianyungang, Jiangsu, China. All the experiments were conducted as per Helsinki’s declaration for human volunteers. All subjects gave informed, signed consent to participate in the study by themselves. Ref. No.2016/053/01.

## RESULTS

Compared with before thrombolysis, the indexes of the 2 groups were significantly improved after thrombolysis, and the improvements of FPG, HbAlc, TG and LDL-C in the control group were better than those in the study group (P<0.05). There was no significant difference between the 2 groups in the levels of TC and HDL-C after thrombolysis (P>0.05), as shown in Table-I.

**Table-I:** Comparison of blood glucose and blood lipid between the 2 groups.

Group	Time	FPG (Mmol/L)	Hbalc (%)	TC (Mmol/L)	TG (Mmol/L)	HDL-C (Mmol/L)	LDL-C (Mmol/L)
Control Group	Before	4.6±0.8	4.6±0.6	4.2±0.9	1.8±0.6	1.1±0.8	2.5±0.7
(N=65)	After	3.2±0.4	2.7±0.3	2.8±0.7	1.1±0.5	0.8±0.4	1.9±0.5
Study Group	Before	7.3±2.7	7.8±1.5	4.2±0.8	2.4±1.2	1.1±0.6	3.1±0.6
(N=70)	After	5.6±1.3	5.4±1.4	2.7±0.6	1.9±0.6	0.9±0.7	2.2±0.4
t/P Control Group	---	8.125/0.000	11.942/0.000	7.118/0.000	5.445/0.000	4.679/0.000	5.081/0.000
t/P Study Group	---	7.457/0.005	6.322/0.000	8.464/0.000	4.978/0.012	4.026/0.005	7.143/0.000
t/P Between Groups After- Thrombolysis	---	9.138/0.000	10.379/0.000	1.054/0.183	7.479/0.000	1.632/0.074	4.992/0.003

The 24h MBG, SDBG and MAGE in the study group were higher than those in the control group, and the difference was statistically significant (P< 0.05), as shown in Table-II.

**Table-II:** Comparison of dynamic glucose parameters and detection indicators between the 2 groups (X̄, mmol/L).

Group	Case	24h MBG	SDBG	MAGE
Control Group	65	5.7±1.6	2.3±0.6	3.4±0.8
Study Group	70	8.5±2.3	3.1±0.4	4.5±0.6
T ---	6.584	9.301	7.902	
P ---	0.000	0.000	0.000	

In the study group, when the blood glucose was less than 6.0mmol/L before thrombolysis, the lowest effective rate after 24h thrombolysis was 33.3%, and when the blood glucose was ranging from 7.0 to 9.0mmol/L, the highest effective rate after 24h thrombolysis was 73.9%, and with the gradual increase of blood glucose, the effective rate after 24h thrombolysis decreased gradually. The effective rate after 24h thrombolysis also decreased gradually with the increase of HbAlc value, it reached the highest value of 64.4% at HbAlc <6.0mmol/Lad the lowest value of 25% at HbAlc >7.0mmol/L, as shown in Table-III.

**Table-III:** The effect of fast blood sugar and HbAlc before thrombolysis on the efficacy at 24h after thrombolytic therapy in the study group.

Index		Case	Age (Years)	Disease Time (h)	Effective (Case)	Invalid (Case)	Effective Rate (%)
BG before thrombolysis (mmol/L)	<6.0	12	65.4±6.2	2.8±1.2	4	8	33.3
	6.0-7.0	20	65.1±5.3	2.8±0.7	11	9	55.0
	7.0-9.0	23	64.2±6.1	3.1±1.3	17	6	73.9
	9.0-11	5	65.5±7.4	3.5±0.8	3	2	66.7
	>11.0	10	66.1±5.5	2.8±1.1	5	5	50.0
HbAlc before thrombolysis (mmol/L)	<6.0	45	65.1±5.4	2.8±1.1	29	16	64.4
	6.0-6.5	12	64.6±5.3	2.7±1.5	6	6	50.0
	6.5-7.0	5	65.4±4.7	3.5±1.2	2	3	40.0
	>7.0	8	65.9±4.8	3.2±1.4	2	6	25.0

Compared with the control group, the MHSS score and MRS score were higher and the Barthel index after thrombolysis was lower in the study group with the difference being statistically significant (P<0.05), as shown in Table-IV. The five months mortality rate after thrombolytic therapy was 12.9% (9/70) in the study group and 10.8% (7/65) in the control group, with no significant difference between the two groups (X^2^=1.085, P=0.316).

**Table-IV:** Comparison of NIHSS, MRS and Barthel index scores between the 2 groups before and after treatment.

Group	Case	NIHSS Score		MRS Score		Barthel Index	

		Before	After	Before	After	Before	After
Control	65	17.32±5.72	9.15±4.33	5.78±0.71	1.76±0.45	27.13±7.06	73.58±9.75
Study Group	70	17.28±5.69	13.24±4.36	5.76±0.65	3.13±0.35	27.15±7.12	50.34±6.28
t/x^2^	---	0.367	5.009	0.941	7.615	0.207	8.046
*P*	---	0.225	0.016	0.378	0.014	0.318	0.011

The incidence of intracranial hemorrhage after thrombolytic therapy was higher in the study group than in the control group with the difference being not statistically significant (P>0.05), but the revascularization and prognosis of the study group were poorer in the study group than in the control group (P<0.05), as shown in Table-V.

**Table-V:** Comparison of intracranial hemorrhage and prognosis between the two groups [n (%)]

Group	Case	Intracranial Hemorrhage	Good Revascularization	Poor Prognosis
Control Group	65	3 (4.6)	46 (70.8)	36 (55.4)
Study Group	70	4 (5.7)	25 (35.7)	25 (35.7)
*x*^2^	---	1.015	9.842	6.337
*P*	---	0.352	0.001	0.008

## DISCUSSION

Existing clinical and epidemiological studies have shown that^6^-^7^ diabetes mellitus is an independent risk factor for ischemic stroke. The relevant mechanism is described as follow^8^-^9^ one description is the fact that the vascular endothelial cells of diabetic patients are prone to functional and structural damage; and the other is that high glucose condition gives rise to disorder of lipid metabolism, the above reasons will increase the probability of intravascular thrombosis and the incidence of cerebral infarction. Rt- PA thrombolytic therapy is the most effective treatment for acute cerebral infarction within the time window (<4.5h). It effectively rescues the nerve cells in the cerebral ischemic penumbra with rapid restoration of nerve function by dredging occluded blood vessels and recovering blood supply in cerebral infarct areas. At present, it has been recommended in the guidelines for cerebral infarction treatment in many countries and in recent years has been widely applied in domestic comprehensive hospitals. Today many clinicians still have doubts on the intravenous thrombolytic therapy for patients with type 2 diabetes and cerebral infarction, and according to the current guidelines for the diagnosis and treatment of acute cerebral stroke, diabetes is not the contraindication of rt -PA intravenous thrombolysis, but there is also clean requirement proposed on the patient’s blood sugar. A research has revealed that^10^ persistent hyperglycemia may indicate poor prognosis in patients after thrombolysis, but there is no definite evidence that proves clear relationship between diabetes and rt-PA thrombolysis induced poor prognosis.

The results of this study showed that the levels of FPG, HbAlc, TG and LDL-C in the study group were higher than those in the control group before and after treatment. Blood sugar fluctuation may also be one of the reasons in addition to atherosclerosis of the arteries caused by long-term hyperglycemia. The researchers have found that^11^-^12^, whether in patients with type 2 diabetes or in healthy people, the glucose with excessive fluctuations greatly damages blood vessel endothelium. And the study found that the dynamic blood glucose parameters of 24h MBG, SDBG and MAGE were higher in patients with acute cerebral infarction and type 2 diabetes than in non-type 2 diabetes patients with acute cerebral infarction. Because the patients in the study group had moderately high glucose and big fluctuation, they also had relatively high risks and more serious condition.

The related research has found that^13^ over- high concentration of blood glucose can lead to disorders of glucose metabolism and vascular endothelial cell dysfunction, in which there will be glycosylation of platelet membrane, leading to platelet activation and making it more susceptible to induced aggregation with the production of superoxide ions and increased synthesis of thromboxane compounds, nevertheless, the synthesis of vasodilatation factors like nitric oxide or prostacyclin is correspondingly reduced, more likely to cause thrombosis. The extremely low level of blood sugar can trigger inflammation responses and inflammatory factors activate complement followed by a large increase of lipid deposited in vascular wall, which induces atherosclerosis and increases the risk of ACI by infiltration and aggregation of vascular endothelial cells. The results of this study showed that the effective rate after 24h thrombolysis was highest when the blood glucose was ranging from 7.0 to 9.0mmol/L and before treatment the efficacy of thrombolysis was moderately poorer when the glucose was too high or too low, and when the blood glucose was less than 6.0mmol/L before thrombolysis, the effective rate after 24h thrombolysis was lowest, only 33.3%. These suggest that different concentrations of pre thrombolytic blood glucose have influences on the efficacy of rt-PA intravenous thrombolysis. Brain is one of human organs with strongest metabolism. Blood sugar is the main source of energy for brain cells, whose capacity of sugar reservation is limited, so when the blood sugar is reduced, the energy needed by brain cells is not sufficiently supplied. In the meantime, the nature of cerebral infarction is attributed to the sudden decrease or cessation of local blood flow of supply artery in brain tissues, leading to cerebral ischemia and hypoxia followed by the resulting brain tissue necrosis and softening. Therefore, the reason for the condition that the effective rate at 24h after thrombolysis is lowest when the blood glucose before thrombolysis is under 6.0mmol/L may be due to the insufficient supply of energy needed by the brain, which, resulting from low blood glucose fails to realize energy supply during rt-PA thrombolysis and eventually causes poor effect.

The results also showed that the efficiency rate after 24h thrombolysis decreased gradually with the increase of HbA1c value, reaching the highest value at HbAlc <6.0mmol/L and the lowest at HbA1c >7.0mmol/L. HbA1c is a product of irreversible binding of hemoglobin in red blood cells with blood glucose. It is directly proportional to blood glucose concentration and can reflect the blood glucose control which is not affected by temporary fluctuation of the concentration. It has been proposed in a number of clinical studies that 14-15 when the HbA1c level increases, more hemoglobin turns to HbA1, leading to reduction of hemoglobin and lower efficiency of oxygen transport. So, the increase of HbA1c leads to insufficient energy supply to the brain, leading to the failure of enough energy supply during rt-PA thrombolysis and poor treatment effect.

This study may be a reply to the suggestions of Shihab et al (2015) who documented that controlled trials are needed to determine whether acute correction of hyperglycemia can improve outcomes after thrombolysis.^16^

The results also showed that there was no significant difference between the 2 groups in the scores of various indexes before thrombolysis, but after thrombolysis, the scores of NIHSS and MRS were higher and the Barthel index was lower in the study group compared with the control group. It can be concluded that after thrombolytic therapy the patients without type 2 diabetes mellitus have better recovery than those with, consistent with the conclusion of previous studies. Moreover, those accompanied by type 2 diabetes have higher risk of cerebral hemorrhage with poorer recanalization and prognosis by contrast.

### Limitations of this Study

However the main drawback of intravenous thrombolysis is symptomatic intracerebral hemorrhage which is the limitation of this study as there was no study to determine the predictive value of parameters of glycosylated hemoglobin.

To sum up, in the course of intravenous thrombolytic therapy for patients with acute cerebral infarction and type 2 diabetes, the level of HbAlc affected the curative efficacy in patients, the higher the level, the poorer the efficacy, and to control the blood glucose within a certain range (7.0-9.0mmol/L) before thrombolysis was beneficial to enhance the effect of static thrombolysis.

### Declaration of interest

All authors reach an agreement and declare to have no competing interests.

### Authors’ Contributions

**ZZ, MQ:** Designed the study, analyzed data and revised the manuscript.

**ZG:** Selection of patients, randomization and prepared the manuscript.

**PZ, LL:** Revised the manuscript, participated on data analysis.

**JC:** Responsible for collecting data and postoperative clinical assessment and revised the manuscript. All authors have approved the final version to be published.
